# Persistence of butterfly populations in fragmented habitats along urban density gradients: motility helps

**DOI:** 10.1038/hdy.2017.40

**Published:** 2017-08-09

**Authors:** E Rochat, S Manel, M Deschamps-Cottin, I Widmer, S Joost

**Affiliations:** 1 Laboratory of Geographic Information Systems (LASIG), School of Architecture, Civil and Environmental Engineering (ENAC), Ecole Polytechnique Fédérale de Lausanne (EPFL), Lausanne, Switzerland; 2 Ecole Pratique des Hautes Etudes, PSL Research University, Centre National de la Recherche Scientifique, Université de Montpellier, Université Paul-Valéry Montpellier, Institut de Recherche pour le Développement, UMR CEFE 5175, Montpellier, France; 3 Aix Marseille University, IRD, Laboratoire Population Environnement Développement, Marseille, France; 4 Swiss Academy of Sciences SCNAT, Swiss Biodiversity Forum, Bern, Switzerland; 5 Urban and regional planning community (CEAT), School of Architecture, Civil and Environmental Engineering (ENAC), Ecole Polytechnique Fédérale de Lausanne (EPFL), Lausanne, Switzerland

## Abstract

In a simulation study of genotypes conducted over 100 generations for more than 1600 butterfly’s individuals, we evaluate how the increase of anthropogenic fragmentation and reduction of habitat size along urbanisation gradients (from 7 to 59% of impervious land cover) influences genetic diversity and population persistence in butterfly species. We show that in areas characterised by a high urbanisation rate (>56% impervious land cover), a large decrease of both genetic diversity (loss of 60–80% of initial observed heterozygosity) and population size (loss of 70–90% of individuals) is observed over time. This is confirmed by empirical data available for the mobile butterfly species *Pieris rapae* in a subpart of the study area. Comparing simulated data for *P. rapae* with its normal dispersal ability and with a reduced dispersal ability, we also show that a higher dispersal ability can be an advantage to survive in an urban or highly fragmented environment. The results obtained here suggest that it is of high importance to account for population persistence, and confirm that it is crucial to maintain habitat size and connectivity in the context of land-use planning.

## Introduction

During the Anthropocene and particularly the last five decades, human population growth and migration have led to an increased demand for housing, transport and infrastructure, leading to a large expansion of cities and to a growing impact of human activities on the environment (Steffen *et al.*, 2007; EEA, 2016). The land-use transformation into dense built-up areas, associated with the intensification of agriculture practices, is mainly responsible for a loss and degradation of natural habitats (Antrop, 2000; EEA, 2016), which is an important cause of endangerment of many animal and plant species (Czech *et al.*, 2000; Wood and Pullin, 2002; Dirzo and Raven, 2003). For example, the change in agriculture practices has been associated with a decline of farmland birds across Europe between 1990 and 2000 (Donald *et al.*, 2006), and habitat loss associated with land-use changes have often been reported to be an important cause of the global decline of amphibian populations (Collins and Storfer, 2003; Cushman, 2006).

In addition, due to landscape fragmentation, habitats of many species become divided by impervious surfaces (roads, buildings and so on.) or other human-influenced areas (cropland, recreational areas and so on.), which reduced natural habitat size and therefore population size too (Fahrig, 2003). In addition, functional connectivity (that is, the movement of individuals among patches) is also affected, subsequently influencing gene flow and reducing genetic diversity (Hitchings and Beebee, 1997; Fahrig, 2003; Coulon *et al.*, 2006). In the region of Marseille, France, Schoville *et al.* (2013) measured genetic diversity in the butterfly *P. rapae* within four regions along a transect leading from the periphery to the city centre and showed decreased genetic diversity in urban versus nonurban sites. Similarly, Takami *et al.* (2004) studied the genetic diversity of two butterfly species from the genus *Pieris* (*Pieris rapae* and *Pieris melete*) in study areas from Japan and Korea. They showed that the genetic diversity is not directly significantly different in urban areas as compared to rural ones. However, important genetic variations can be observed among seasonal subpopulations in urban areas, whereas it is not the case in rural ones. As a consequence, when considering seasonal subpopulations, the genetic diversity in cities is reduced as compared to rural environments. As the reduction of population size and genetic diversity induces a higher risk of inbreeding (Bonte *et al.*, 2012) and a lower adaptive potential (Munshi-South *et al.*, 2016), this can make species more vulnerable to extinction, particularly if exposed to further environmental changes (Allendorf and Leary, 1986).

However, urban habitats can also harbour self-sustaining populations of native (and exotic) species (Kowarik, 2011) that are able to adapt to the human-influenced environment or even take advantage of the proximity with humans that may provide food sources, reduce the presence of wild predators or provide new refuges (McKinney, 2002; Shochat *et al.*, 2010). Indeed, organisms can adapt to anthropogenic fragmentation, either morphologically or behaviourally (Cheptou *et al.*, 2017). For instance, Evans *et al.* (2009) showed that the forest-specialist blackbird, *Turdus merula*, was able to colonise and adapt to urban environments. Despite a little reduction in expected heterozygosity within urban populations of this blackbird, no reduction of observed heterozygosity was noticed and no evidence of genetic differentiation was highlighted between urban and rural populations. Similarly, Lourenço *et al.* (2017) showed no significant differences in genetic diversity between urban and rural populations of salamanders in the municipality of Oviedo (Spain). Previous studies showed that some factors could facilitate the adaptation to urban environment, notably a higher dispersal ability or a lower habitat selectivity (Turin and den Boer, 1988; Maes and Van Dyck, 2001; Wood and Pullin, 2002; Takami *et al.*, 2004). However, estimating the impact of urbanisation and fragmentation on species remains complex. Moreover, the direct observation of species and the measure of their genetic diversity may be difficult due to the restricted number of individuals still present in urban or highly fragmented environments. Nevertheless, the identification of plant and animal species endangered by the ongoing fragmentation of habitats is essential in order to promote more sustainable urbanisation processes or conservation strategies in the future. In such situations, simulations are a valuable tool to analyse the landscape fragmentation, the structural and functional connectivity between habitats and the impact of such landscapes on the genetic diversity of various species.

In this context, our study combines empirical and simulated data to analyse the impact of landscape elements on the genetic diversity and on the persistence of butterfly populations in the region of Marseille, France. Butterflies, as airborne species that require open spaces to fly, are constrained in their movements in fragmented and human-influenced landscapes and are therefore interesting model species. In this study, we aim for the following: (1) to analyse the spatiotemporal evolution of the genetic diversity and population persistence as a function of the urbanisation level from simulated genetic data (500 single nucleotide polymorphism (SNP) loci over 100 generations) for a butterfly species with high dispersal capacity (*P. rapae*); (2) to study the impact of a simulated reduction of the dispersal ability on this evolution; and (3) to compare spatially explicit simulation results with measured genetic diversity of *P. rapae* populations, estimated from an empirical data set (366 amplified fragment length polymorphism (AFLP) markers, Schoville *et al.*, 2013).

## Materials and methods

### Study area

The study area is centred on the region of Marseille, south-east France. With 855 393 inhabitants in 2013, Marseille is the second most populated French municipality, after Paris (source: www.insee.fr, population census, 2013). In order to capture the landscape heterogeneity of this region, we combined vector and raster data describing the land cover of the Marseille area (IGN BD Carto 2004, SPOT 5 2004, Lizée *et al.*, 2011) and produced a land cover classification map of eight classes (spatial resolution: 10 m): buildings (divided into four subclasses as a function of the building height), roads and other impervious surfaces, mixed surfaces (artificial and natural), grasslands, parks, forests, open areas (mainly not vegetated) and water.

**Figure 1:**
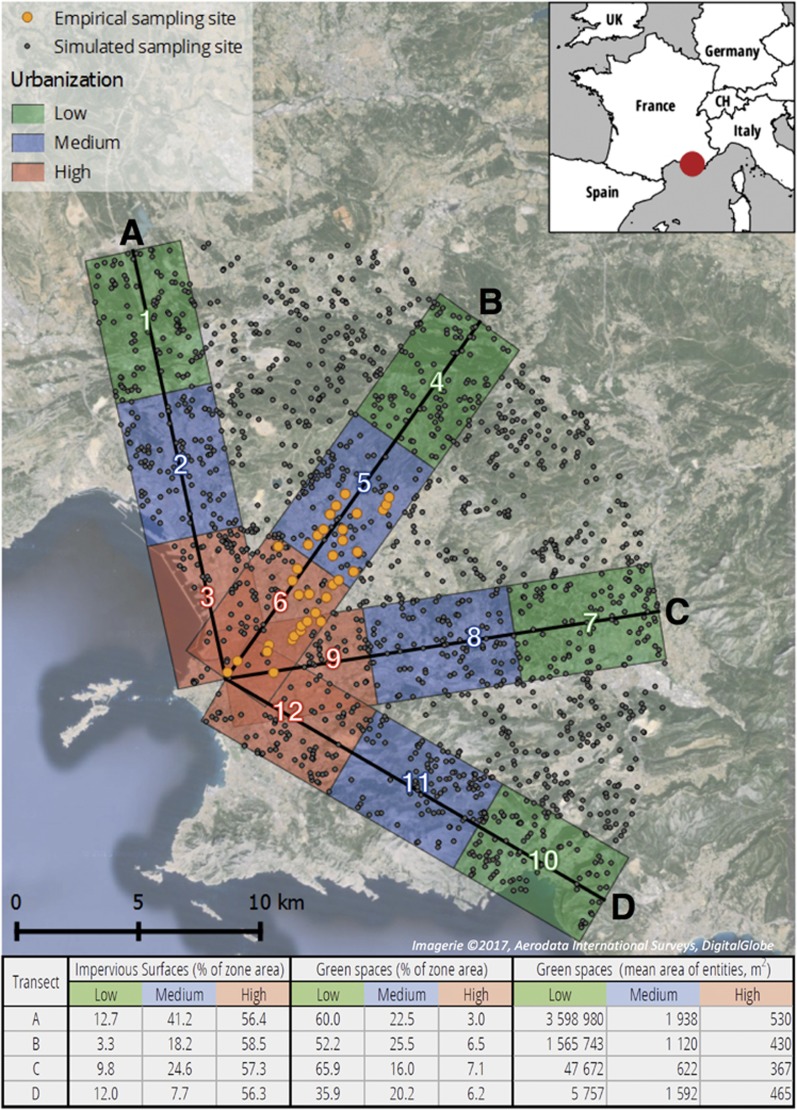
Four transects along urbanisation gradients leading from the city centre of Marseille (Vieux-Port) to more rural areas. Along each transect, three zones were delimited and represent different urban densities (high, medium and low). For each zone, the table indicates the percentage of land covered by impervious surfaces or green spaces, as well as the mean surface of green spaces' entities. The sampling sites for the simulation of butterfly populations and genotypes were then randomly assigned to potential habitats within the 12 zones (100 sites per zone). Transect B contains the empirical sampling sites (yellow points) of *P. rapae* used by Schoville *et al.* (2013).

To simulate the impact of urbanisation on the evolution of populations and genotypes, we then focused on 12 equally sized spatialized areas categorised into three levels of urban densities (low, medium and high). These study areas were defined along four transects, each of 18 km in length and 4 km in width, leading from the Vieux-Port of Marseille (city centre) to the suburbs and therefore showing a high urbanisation gradient from densely populated urban to more natural areas. As a function of the urban densities, we divided lengthwise each transect into three parts: high urban density being the first 6 km along the transect from the city centre (red-coloured zones in Figure 1), medium urban density from 6 km to 12 km (blue-coloured zones) and low urban density from 12 km to 18 km (green-coloured zones). We thus defined 12 rectangular areas (4 km width, 6 km length), partially overlapping downtown (Figure 1).

In order to compare results from simulations and empirical data, we used an empirical genetic data set for the butterfly species *P. rapae* (Schoville *et al.*, 2013). Consequently, one of the simulated transects (Transect B in Figure 1) was chosen to correspond to the sampling direction and sites of the empirical study published by Schoville *et al.* (2013). The three other directions were spatially distributed such as to be representative of the variations in urbanisation level around Marseille.

Based on the land cover map, we then computed the percentage of each land cover classes in the 12 defined areas. The areas with high urbanisation level are characterised by 56–59% of impervious land cover (buildings, roads and other impervious surfaces), 3–7% of green spaces (grasslands and parks), 10–22% of forests and 15–30% of other land cover types (water, mixed surfaces and open areas). The medium urbanised areas contain 8–41% of impervious land cover, 16–26% of green spaces, 19–46% of forests and 17–32% of other land covers. Finally, the areas with a low urbanisation level show 3–13% of impervious land cover, 36–66% of green spaces, 21–37% of forests and 3–28% of other surfaces (Figure 1).

### Resistance map

Based on expert opinion and empirical results, we assessed and assigned a relative resistance value to each class of the land cover classification, as a function of the capacity of *P. rapae* to disperse in each type of land cover (Table 1).

As *P. rapae* is a butterfly species preferring open and sunny vegetation-covered landscapes (Ohsaki, 1979), we attributed the lowest resistance value to green spaces (grasslands and parks, resistance=1). The mean dispersal distance for females of *P. rapae* during their lifetime is about 2 km (Jones *et al.*, 1980). On this basis, we approximated and fixed their maximum dispersal distance in the most favourable land cover class to 4 km. The open areas mainly not covered by vegetation are not a barrier to dispersal but do not offer many foraging possibilities and are therefore less attractive for butterflies than the green spaces (resistance=3). Water surfaces, buildings under two metres of height, roads and other impervious surfaces can still be potentially crossed but are not vegetated and not necessarily open and will therefore probably not be chosen preferentially as dispersal directions (resistance=10). An intermediate resistance has been considered for mixed surfaces, as they can contain each of the previous-mentioned land cover classes in various proportions (resistance=5). Forests are not favourable at all for the dispersal of *P. rapae* (Ohsaki, 1979) and we thus assigned them a high resistance (resistance=20). Finally, buildings constitute barriers to dispersal and received the highest resistance values, increasing as a function of building height (4 categories, resistances of 10, 20, 50 and 450), with the buildings over 10 m in height considered as impossible to cross.

Based on these resistance values, we produced a resistance map by assigning to each pixel of the land cover map the corresponding resistance value. The resulting map shows the dispersal cost through each pixel and was used to compute least-cost path between two sites.

### Habitat

In order to simulate the evolution of populations and genotypes along the four transects, we first defined sites within potential habitat areas for *P. rapae*. To this end, we used the software QGIS 2.14 (function random selection within subsets) to randomly assign 100 sites to potential habitats for *P. rapae* (that is, green spaces) within each of the 12 zones. The number of sites per zone (100) was chosen in order to obtain realistic distances between the sites but to avoid overestimating the number of potential habitats, notably in the city centre. In order to ensure the largest habitats—which are assumed to harbour important butterfly subpopulations—to be represented in the sampling design for the simulations, we applied a stepwise procedure. We first chose to randomly position three sites in each of the green spaces showing an area of at least five hectares (ha), and we then placed one site in each of the green spaces showing an area between 1 ha and 5 ha. All the other sites—required to achieve a total of 100 sites per zone—were randomly positioned within green spaces of at least 200 m^2^ (2 pixels). Due to partial overlapping between the zones (mainly in the city centre), some points were counted for two different areas. We ended up with a total of 1083 sites for simulations, with a median nearest-neighbour distance of about 320 m. In addition, in order to allow for connectivity and potential gene flow among populations from different transects, 550 sites were randomly distributed within green spaces situated between the transects, as illustrated in Figure 1.

### Simulated data

Once the sampling sites were defined, we used the individual-based population genetics model software CDPOP 1.2.21 (Landguth and Cushman, 2010) to simulate the evolution of genotypes over 100 generations. In order to benefit from the possibility offered by the simulations to obtain a high number of genetic markers, but to avoid simulating a data set too different from the empirical data set available (366 AFLP markers), we decided to simulate the evolution of genotypes at 500 diploid biallelic SNP loci.

CDPOP enables the user to define various demographic parameters related to the displacement of individuals between the sites, the mate choice, the breeding with Mendelian inheritance and the mortality of individuals. Here, we started the simulations by considering a uniform distribution of butterflies over the study region, that is, all sites previously defined were assumed to be inhabited by one individual of *P. rapae* at the beginning of the simulations (in total 1633* P. rapae* individuals over the study region). The initial genotypes were randomly assigned. The simulation of dispersal and mating movement between the sites was then based on a cost–distance matrix indicating the cost of dispersing from one site to another. For this matrix, we defined the dispersal cost as the cumulative resistance of the least-cost path, computed using the software Graphab (Foltête *et al.*, 2012) based on the resistance map previously defined. The function used to link the dispersal cost and the dispersal probability has then to be chosen between the four possibilities offered by CDPOP: linear, inverse square, nearest neighbour and random mixing. We chose here the linear one, assuming that the probability to disperse decreases linearly with the increase of the cost. Finally, we specified the maximum dispersal distance. As for the resistance maps, we approximated and fixed the maximum dispersal distance of females of *P. rapae* to 4000 m. The males are less mobile and scarcely dispersed (Ohsaki, 1980), and their maximal dispersal distance was therefore defined to approximately one-third of the one of females (that is, 1350 m).

For breeding parameters, we considered a sexual reproduction that can start from the age 0, with no selfing and no philopatry, both males and females allow to mate multiple times (Bissoondath and Wiklund, 1996) and multiple paternities possible (females can have offspring from multiple males). The number of offspring of *P. rapae* can vary between 300 and 400 eggs with about 99% mortality (Richards, 1940) and was thus simulated using a Poisson's law of parameter *λ* equal to 300, with a birth mortality fixed at 99%. The sex of each individual was set randomly for each generation.

Finally, generations of *P. rapae* can sometimes partially overlap (Ohsaki, 1982), and we therefore fixed the adult mortality to 95%, which keeps the possibility of 5% of the individuals to live for more than one generation. The complete list of parameters used is presented in Supplementary Table 1. In order to increase reliability of our results, we computed five runs of simulations based on these parameters.

In a second step, we computed five additional runs of simulations using exactly the same parameters, except that we reduced the maximal dispersal distance by one half, that is, 2 km for the females and 675 m for the males. This second set of simulations corresponds to the simulation of *P. rapae* with a reduced dispersal ability and enables the analysis of the influence of the dispersal capacity on the evolution of genetic diversity and population persistence.

Our simulations finally produced a data set of 500 SNPs markers for 1633 individuals (1083 situated along 4 transects leading from the city centre to the periphery and divided into three levels of urbanisation, and 550 individuals in between these transects) over 100 generations. The data set is replicated 5 times (5 runs) for *P. rapae* with normal dispersal ability, and 5 times for *P. rapae* with reduced dispersal capacity.

### Genetic diversity and population persistence

Once the genetic data have been simulated over 100 generations, we analysed the level of genetic diversity within each of the 12 zones, based on measures of heterozygosity. A high heterozygosity indicates a lot of genetic variability, whereas a low heterozygosity indicates poor genetic diversity. We used here two indices: the average observed heterozygosity (*H*_obs_) as well as the average expected heterozygosity (*H*_exp_) assuming Hardy–Weinberg equilibrium:


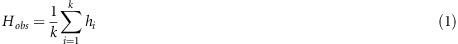


and


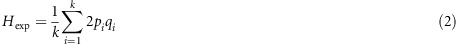


where *h_i_* is the frequency of individuals that are heterozygous for the marker *i*, *p_i_* (respectively *q_i_*) is the frequency of presence of the first (respectively second) allele for the marker *i* and *k* is the total number of markers. The values of these measures range from 0 (no individual heterozygous for any marker, no genetic diversity) to 1 (all individuals are heterozygous for all markers, high genetic diversity). The comparison of the values of these two indices can allow the identification of potential inbreeding. Indeed, when a population is facing high inbreeding, the fraction of heterozygotes observed will be less than what is expected under random mating. The difference between observed and expected heterozygosities can therefore be used to estimate the amount of current inbreeding (Wright, 1949).

During the simulations, habitat sites may become uninhabited, in particular if the cost of reaching sites is too high. As a result, the number of individuals per zone can change over time (starting from an initial value of 100 individuals per zone). We retrieved the number of individuals remaining in each zone at each generation (*N*) and used this number as an estimate of the population persistence in the respective zone.

For both dispersal abilities and for each of the 12 zones, we used the five values resulting from the five simulation runs to compute mean (μ), s.d. (*σ*) and 95% confidence intervals (μ±1.96*σ/√n) for the three parameters (*H*_obs_, *H*_exp_ and *N*) at each generation and we produced plots of their evolution over time. In order to compare the genetic diversity and number of individuals remaining in each zone at the end of the simulations, we also used the 5 values from the 5 simulation runs to compute a one-way ANOVA between the last values (generation 100) of *H*_obs_ (resp. *H*_exp_ and N) in each of the 12 zones (that is, 12 groups, 5 measures per groups). Post-hoc testing was then performed using a Scheffé’s test in order to highlight the significant differences between the zones. All computations were performed using the Matlab R2014b software (functions *anova1* and *multcompare*).

### Empirical data

With the objective to compare the results of the genetic diversity obtained by simulations to an empirical case study, we used a published empirical data set of *P. rapae* sampled in the same study region (Schoville *et al.*, 2013). This data set was composed of 366 AFLP markers for 219 *P. rapae* individuals that were sampled at 41 sites along a 100 km transect going from the Vieux-Port of Marseille to the suburbs. We here used a subset of this data set, containing only the sampling sites present in our study area, which corresponds to 36 sites and 145 individuals (yellow points on transect B, Figure 1). In order to estimate genetic diversity based on this empirical data, for each site we identified the n nearest neighbours (Euclidean distance between sampling points) for n comprised between 3 and 25. We then computed the expected heterozygosity among the individuals from the group of neighbouring sites. As AFLP markers do not allow the distinction between heterozygotes and homozygotes of the dominant allele, we can only measure the frequency of homozygotes of the recessive allele (*f*). Assuming Hardy–Weinberg equilibrium with *p* representing the allele frequencies of the dominant allele and *q* the allele frequencies of the recessive allele, we have *f*=*q*^*2*^ and 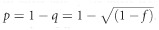. The expected heterozygosity can therefore be expressed as follows:


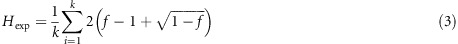


where *k* is the total number of markers.

In order to compare simulated and empirical data, the expected heterozygosity for the simulated data was also computed along the same transect (transect B) by considering for each site the *n* nearest neighbours (*n* between 3 and 25), using formula (2).

## Results

### Observed heterozygosity

**Figure 2:**
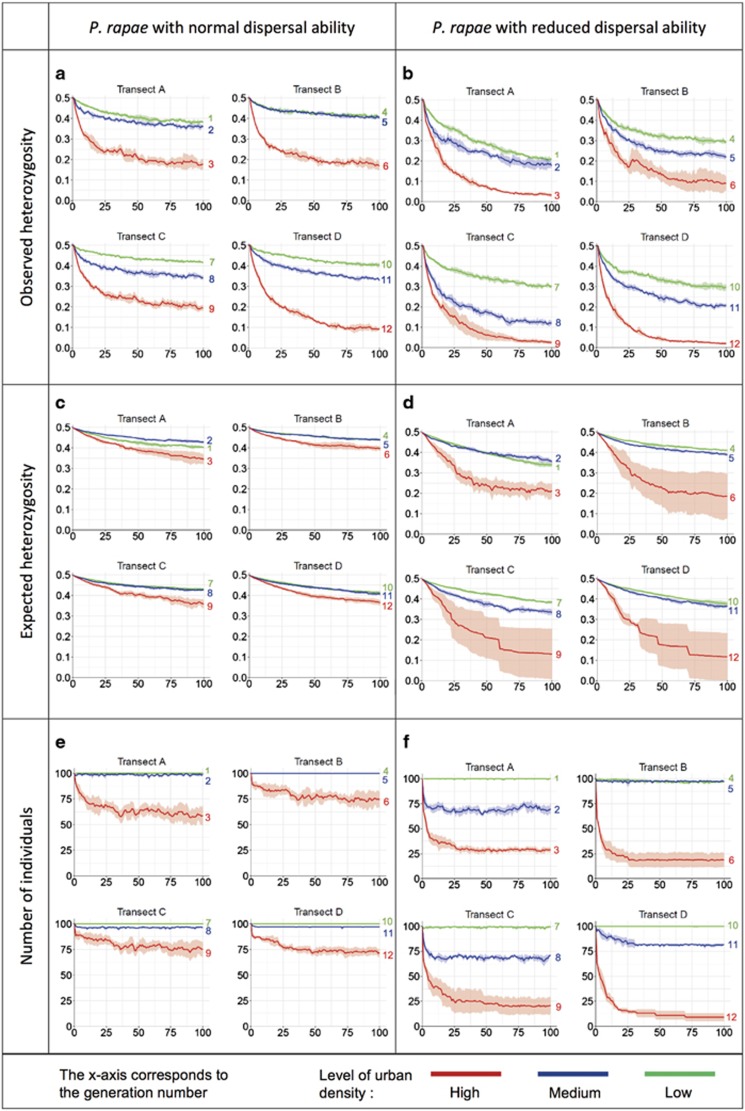
Simulated change over time of the number of individuals (**e**, **f**), and observed (**a**, **b**) and expected (**c**, **d**) heterozygosities within areas of different urbanisation densities. Graphs in the left column show the changes over time for *P. rapae* with normal dispersal ability and in the right column for reduced dispersal ability. For each transect, the green line corresponds to the more rural area (green areas in Figure 1), the blue line to intermediate area (blue areas in Figure 1) and the red line to the city-centre area (red areas in Figure 1). The curves present the average value and the 95% confidence intervals computed on the basis of the five simulation runs.

The change over time of the genetic diversity, as measured by the observed heterozygosity is presented in Figure 2a (*P. rapae* with normal dispersal ability) and 2b (*P. rapae* with reduced dispersal ability). The initial value at generation zero is equal to 0.5, which corresponds to the theoretical maximum value for heterozygosity expected under Hardy–Weinberg equilibrium, resulting from the random distribution of genotypes at the beginning of the simulations. For both dispersal capacities, this value rapidly decreases in all transects and for all levels of urbanisation.

In the more rural areas (green lines), a loss of 6–7% of the initial heterozygosity can be observed after 10 generations for *P. rapae* with normal dispersal ability, but the decline then stabilises and more than 75% of the initial heterozygosity level is still present after hundred generations (Table 2). Similar evolution can be observed with the reduced dispersal, with however a more pronounced decline (15–19% lost after 10 generations, 40–58% at generation 100). The results of the ANOVA and Scheffé’s tests are presented in Supplementary Tables 2 and 3. Scheffé tests computed on the values reached at the end of the simulations indicate that with reduced dispersal, the loss of observed heterozygosity in area 1 (transect A, −58.3%) is significantly higher (*P*-values <10^−6^) than that in the other areas with a similar urbanisation level (maximum 41.4% lost). This area is also showing the highest loss among the areas of low urbanisation levels with the normal dispersal ability.

In the areas with medium urbanisation (blue lines), a loss of 9–15% of the initial heterozygosity can be observed with the normal dispersal ability after 10 generations and of 20–32% at the end of the simulation. Once again, the decline is more pronounced for the reduced dispersal, where the loss already reached 25–36% at generation 10 and 56–76% at the end of the simulations. The values of observed heterozygosities in these medium urbanised areas (blue lines) are generally lower than those in the areas with low urbanisation (green lines). However, for some transects, the values are close and the confidence intervals sometimes overlap (transects A and B for normal dispersal, transect A for reduced dispersal). The Scheffé’s tests computed on the values at generation 100 indeed indicate significant differences between the values of areas with low and medium urbanisation only in transects C and D for the normal dispersal (*P*-values <10^−6^) and in transects B, C and D for reduced dispersal (*P*-values <10^−4^).

Finally, for highly urbanised areas (red lines), more than 30% of the initial observed heterozygosity is already lost after 10 generations with the normal dispersal and the decline continues until the end of the simulations, after hundred generations, where only 28–40% of the initial observed heterozygosity remains (Table 2). With the reduced dispersal, the level of observed heterozygosity remaining after hundred generations is dramatically low (between 0.1 and 0.15, representing only 4–28% of the initial value). For both dispersal abilities, Scheffé’s tests indicate that the values reached at generation 100 in all highly urbanised areas are significantly lower than those in all areas with medium or low urbanisation (*P*-values <10^−7^). We can also notice that with the normal dispersal ability, the value of observed heterozygosity reached at generation 100 in area 12 (transect D, −96.2%) is significantly lower than that in the other highly urbanised areas (*P*-values <10^−6^). This area is also the one presenting the highest loss of observed heterozygosity with the reduced dispersal ability (−96.2%).

### Expected heterozygosity

Like the observed heterozygosity, at the beginning of the simulations the expected heterozygosity is equal to 0.5 in all transects for *P. rapae* with normal dispersal ability (Figure 2c) and reduced dispersal ability (Figure 2d). Over time, a decrease can be observed in all transects and all areas, but this decline is less pronounced than that for the observed heterozygosity, especially with the normal dispersal ability. Indeed, with this dispersal, the highest loss of expected heterozygosity after hundred generations is of 30.6% (Table 2, transect A, highly urbanised) whereas it was of 82.1% for the observed heterozygosity (Table 2, transect D, highly urbanised). With the reduced dispersal, the decreases are more pronounced, especially in the highly urbanised areas, and the values are also less stable between the simulation runs, which is highlighted by the much larger confidence intervals.

When comparing the various levels of urbanisation, no significant difference can be highlighted for any transect between the areas of low or medium urbanisation, when considering the values of expected heterozygosity at generation 100 (*P*-values >0.5) with either dispersal abilities. The values of expected heterozygosity in the more rural areas (green lines) even occasionally drop below the value of the corresponding intermediate areas (blue lines), notably in transect A. With the normal dispersal ability, the differences between highly urbanised areas and the other levels of urbanisation are small but nevertheless significant (*P*-values <0.03), except for area 6 (transect B). With the reduced dispersal, only the areas 9 and 12 (transects C and D) show significantly lower values at generation 100 as compared to the other levels of urbanisation (*P*-values <0.05).

### Persistence of populations

For *P. rapae* with normal (Figure 2e) and reduced (Figure 2f) dispersal abilities, the results show that the number of individuals in the less urbanised areas (green lines) remains stable throughout the study period, for all transects. Indeed, with the normal dispersal, hundred individuals are present at all time in these areas (Figure 2e, green lines), whereas with the reduced dispersal (Figure 2f, green lines), a small loss can be noticed in half of the transects (B and C), but this loss is only of 3 individuals at maximum (Table 2).

For the areas with a medium urbanisation (blue lines), with the normal dispersal (Figure 2e) the number of individuals slightly decreases but then remains also stable through time, with more than 96 individuals present at all time in all transects. However, with the reduced dispersal (Figure 2f), a noticeable reduction in the number of individuals (18–30 individuals) can be observed in transects A, C and D, whereas in transect B the loss of individuals is very small (3 individuals at maximum). Scheffé’s tests indicate that the values reached at generation 100 are not significantly different between low- and medium urbanised areas for the normal dispersal ability (*P*-values >0.9). However, with the reduced dispersal, the values are significantly different (*P*-values <0.001) except in transect B.

Finally, for the highly urbanised areas (red lines), with the normal dispersal, the number of individuals (Figure 2e) rapidly drops from 100 to <85 during the first 10 generations, and this decline is even more severe with the reduced dispersal (Figure 2f) for which <40 individuals are present after 10 generations. After approximately 20 generations, the number of individuals in these highly urbanised areas stabilises for both dispersal abilities. With the normal dispersal, the stabilisation occurs in around 60 (transect A) to 75 individuals (transects B, C and D), whereas with the reduced dispersal it is much lower (9 individuals in transect D to 30 individuals in transect A). Once again, the Scheffé’s tests indicate that the values reached at the end of the simulations in the highly urbanised areas are always significantly lower than those in the ones with medium or low urbanisation, with both dispersal abilities (*P*-values <2 × 10^−4^).

### Simulated versus empirical results

**Figure 3:**
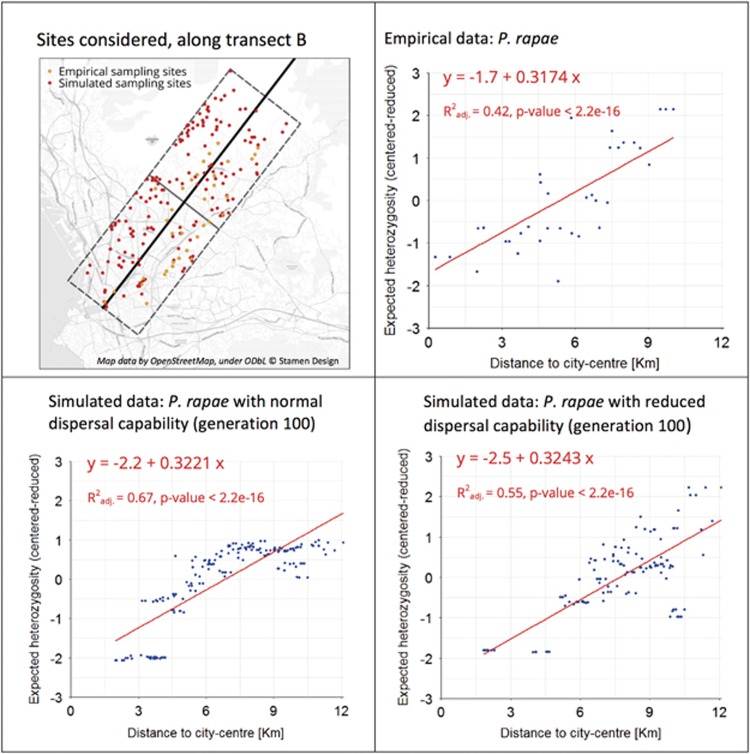
Expected heterozygosity computed for each site considering the five nearest neighbours, as a function of the distance to the city centre. For both simulated and empirical data, the values of heterozygosity have been standardised. Note that the absolute values are not directly comparable as the values of expected heterozygosity computed on the empirical data sets (366 AFLP) range from 0.07 to 0.18, whereas from simulated data (500 SNP) they are comprised between 0.012 and 0.42 (normal dispersal ability) or between 0.005 and 0.37 (reduced dispersal ability). The linear fit and the statistics were obtained using the function lm in R.

For both the empirical and simulated data, we can observe a significant increase of expected heterozygosity with increasing distance from the city centre (Figure 3). Moreover, the linear regressions fitted on the centred-reduced values show slopes that are very close for all data sets. When comparing the absolute values of expected heterozygosity obtained with the simulated and empirical data sets, one can notice that the range of values obtained from the empirical data set is much smaller than the one from simulations. Indeed the values of expected heterozygosity computed on the empirical data set (366 AFLP) range from 0.07 to 0.18, whereas from simulated data (500 SNP) they are comprised between 0.012 and 0.42 (normal dispersal ability) or 0.005 and 0.37 (reduced dispersal ability). However, despite these differences in the absolute values, the same patterns with respect to urban density are observed in both the simulated and empirical data.

Figure 3 presents the evolution of the expected heterozygosity as a function of the distance to the city centre for the computations performed considering for each site the 5 nearest neighbours. The results for the other numbers of neighbours (3–25) are not presented, but they lead to the same conclusions.

## Discussion

### Potential negative impact of anthropogenic fragmentation

Results of this study illustrate that highly urbanised areas show a lower genetic diversity for butterflies, measured by both the observed and expected heterozygosities. These areas are characterised by a high percentage of impervious land cover (>55%), a low percentage of green spaces (<8%) and a reduced surface of green spaces entities (<600 m^2^; Figure 1). In these conditions, the loss of genetic diversity observed can be explained both by the reduction in population size due to the loss of habitats and to their smaller size, and also to the limited connectivity due to the dispersal barriers caused by impervious surfaces. Indeed, during the simulations, when the resistance of the landscape is important, the cost of moving to other habitats becomes too high and eventually individuals can only reach very few congeners to reproduce. In such situations, gene flow is significantly reduced, which ultimately leads to a decline of genetic diversity. This decline has also been highlighted by the analysis of the empirical data available in the study region for *P. rapae*, which shows a decrease of the expected heterozygosity towards the city centre. Moreover, several previous studies have highlighted a similar negative influence of urban environment on dispersal (Schtickzelle and Baguette, 2003; Schtickzelle *et al.*, 2006; Dubois and Cheptou, 2017), gene flow (Keyghobadi *et al.*, 2006) and genetic diversity (Williams *et al.*, 2003; Takami *et al.*, 2004). Nevertheless, the rapidity of the decline presented here with the simulations should be interpreted with caution. Indeed, in real environments, populations are generally much larger than 100 individuals, and the reduction in genetic diversity may therefore take more time than what is presented here. However, the aim of the analysis was not to determine the time required to reach a given level of genetic diversity, but to show that simulated as well as empirical data indicate that the genetic diversity of urban populations is significantly reduced as compared to the diversity of populations living in more rural areas. Moreover, the results show that the level of observed heterozygosity is generally lower than the level of expected heterozygosity, especially in the highly urbanised areas. This difference highlighted a potential inbreeding for the populations concerned, which is a cause of extinction risk for butterfly populations (Saccheri *et al.*, 1998; Nieminen *et al.*, 2001).

Results of the simulations also highlighted a decrease in the number of *P. rapae* individuals over time, especially in highly urbanised areas. This decrease suggests that the persistence of populations is threatened in urban environments. For the simulated data, a potential site may become unoccupied over time if the individuals living there are no longer able to find a congener to reproduce. Indeed, in this extreme case, individuals are isolated and the population is doomed to extinction. This negative impact of urban environment on the persistence of populations has already been highlighted in other urban areas and for other species (Maes and Van Dyck, 2001; Wood and Pullin, 2002; Fattorini, 2011). However, once again, when considering the number of generations after which a site potentially becomes uninhabited, the results of the simulations should be interpreted with caution. In reality, depending on the initial size of the population present in each habitat, the real extinction may take more time than what is shown here. Nevertheless, independently of the exact time required for extinction, the simulations we processed highlighted potential habitats in which populations are particularly vulnerable due to the lack of connectivity with their neighbouring habitats.

Our study was conducted in an environment, in which the fragmentation and reduced habitat size was mostly due to a high level of urbanisation. However, the negative impact on genetic diversity and population persistence highlighted here can also be observed in nonurban environments facing important fragmentation and reduction of habitat size. For example, Fountain *et al.* (2016) studied museum samples of the Glanville fritillary butterfly and showed that a decline in genetic diversity was preceding the extinction of the populations in the mainland of Finland mainly due to fragmentation and loss of suitable meadows. Similarly, loss of genetic diversity due to fragmentation and associated lack of connectivity has been highlighted for the prairie chickens in Wisconsin (Johnson *et al.*, 2004), for the alpine chipmunk in Yosemite National Park (Rubidge *et al.*, 2012) or for a tropical rain forest tree in Costa Rica (Hall *et al.*, 1996).

Finally, we note that the impacts highlighted in this study are not relevant for all species living in urban environments. Even though similar evolutions could be most probably observed for other butterfly species that are similarly constrained in their dispersal in urban environments, other species may not be negatively impacted by urbanisation, or less impacted, as previously mentioned in the introduction.

### Differences among transects: land cover and barriers to dispersal

As regard to the areas with low urbanisation, the highest loss of genetic diversity or number of individuals is generally observed in transect A. This area of transect A is characterised by a high percentage of green spaces (60%) and a large average surface for these entities (>3 km^2^) likely to be favourable for the species studied. However, this area also shows the highest percentage of impervious surfaces (12.7%), which could explain its disadvantage as compared to other regions of the periphery.

For the areas with medium urbanisation, transect C often seems to be the most negative, especially for the species with the lower dispersal ability. This could be explained by the lower percentage of habitat areas (grassland and parks) in this region and also to the smaller average surface of habitat entities (622 m^2^), which indicates a higher fragmentation.

Finally, the highly urbanised areas of transect D often appear to be less favourable even if its land cover does not seem to be very different from the other transects. However, this transect is characterised by the lowest percentage of green spaces in the periphery, which is due to a quite high percentage of impervious surfaces (12%) but also a high percentage of water (11.8%) and forest (24.4%). These barriers may reduce the gene flow from the periphery to the city centre and therefore threaten the viability of the populations of the city centre. This shows that the fragmentation of the less urbanised suburb areas can also have a noticeable importance on the decrease of genetic diversity and population persistence of the urban populations.

Finally, we note that in our case for all parameters (expected and observed heterozygosity and number of individuals), the differences highlighted between the transects (differences at generation 100: 0–27.4%, 12.9% in average) remain minor relative to the differences observed between the three levels of urbanisation (differences at generation 100 between low and high urbanisation level: 9–91%, 43.7% in average).

### Dispersal capacity in urban landscapes

When comparing the respective behaviours of *P. rapae* and of a butterfly with a reduced dispersal ability, results show that the reduction of genetic diversity is much more pronounced for the less mobile species and that the persistence of the latter populations is also more threatened. This underlines that a higher dispersal capacity may be an advantage for species living in urban environments, which had already been highlighted by previous studies (Maes and Van Dyck, 2001; Wood and Pullin, 2002; Duplouy *et al.*, 2013). Indeed, a higher dispersal capacity results in the ability to disperse over longer distances but also to use various dispersal modes facilitating the crossing of barriers present in urban landscapes. For example, plants pollinated by many insects may be only moderately impacted by urban fragmentation (Culley *et al.*, 2007). Similarly, species that can benefit from human-mediated dispersal (attachment to clothes, vehicles, shoes, soil movements and so on.) may be particularly adapted to urban landscapes (Banks *et al.*, 2015; Egizi *et al.*, 2016). Conversely, species with only one dispersal mode such as butterflies or plants pollinated only by specific insects may be more strongly influenced by urbanisation and endangered by the induced fragmentation (Cheptou *et al.*, 2017).

### Relevance of simulations

This study’s results illustrate advantages of combining simulated with empirical data in landscape genetics. Indeed, empirical genetic data reflect the current state of the genetic composition of populations, influenced by potentially unknown evolutionary processes in the past. However, the collection of such data might be particularly expensive and time-demanding. In this context, simulations may offer many advantages (Epperson *et al.*, 2010).

First, as shown in this study, the use of simulated data allows for the extension of the analysis over a larger study area including zones showing a diversity of urbanisation levels, and over a defined period of time. This notably makes it possible to compare several transects and to highlight local differences across the metropolitan area of Marseille, while empirical data were restricted to a single transect.

Second, the simulations enable the study of a species with a lower dispersal distance, for which it may be difficult to collect samples due to its limited presence in urban environments. Here, the simulations permitted in particular to emphasise the threat that dense urban areas constitute for low dispersal species compared to species with a higher dispersal capacity.

Finally, simulations allow the consideration of a larger genetic data set, here based on 500 SNPs as compared to the 366 AFLPs constituting the empirical data set. This can be particularly interesting in a context of high sequencing cost and since results may change according to the genetic data used (Landguth *et al.*, 2012).

However, simulations often require subjective, expert-based assumptions to be formalised (resistance values, populations’ parameters and so on.) resulting in the injection of uncertainty in the results obtained. This is an important reason why the combination with empirical data is particularly powerful since the latter provide landmarks to relieve the uncertainty mentioned above and enabling a complete analysis and a more confident interpretation of the results.

### Sustainable land-use planning

Our results show that butterfly species can be strongly threatened in dense urban areas, highly fragmented environment or other human-influenced areas. For these species, in order to conserve and promote genetically stable and diverse populations, it is important to (1) preserve or restore suitable habitats and (2) maintain or increase the connectivity among them in order to allow dispersal also for species with limited dispersal capacities. As increasing the connectivity by the creation of dispersal corridors may be difficult to achieve due to the numerous constraints of urban or highly fragmented environments, the creation or preservation of stepping stone habitats is promising and of special importance (Bierwagen, 2006; Serret *et al.*, 2014). In this context, the use of landscape genetic methods to assess the impact of landscape features on gene flow is a key step in designing functional ecological networks aiming to preserve genetic diversity and therefore biodiversity (Baguette *et al.*, 2013). Genetic analyses are powerful methods to estimate species’ dispersal processes (Stevens *et al.*, 2010), to assess adaptive ability (Munshi-South *et al.*, 2016) and also to directly provide information about persistence of populations, which is essential to promote and plan for more sustainable land-use strategies. In urban areas, the preservation of biodiversity is also key to favour a better quality of life for the residents, including well-being related to better health conditions (Maller *et al.*, 2006). For this reason, it is of paramount importance that urban authorities and planners adapt the way they design dense city centres in particular, to identify the potential consequences on native species, and to favour the insertion of connected habitats.

## Data Archiving

All simulated data have been submitted to Zenodo: doi:10.5281/zenodo.215722.

## Figures and Tables

**Table 1:** Resistance values for the various land cover classes, used to model the dispersal of the *P. rapae* butterfly in the region of Marseille, France

*Land cover class*	*Resistance of a 10-m pixel*	*Maximal dispersal distance in the land cover class (m)*
Green spaces (grasslands and parks)	1	4000
Open areas (mainly not vegetated)	3	1300
Mixed surfaces (artificial and natural)	5	800
Water	10	400
Roads and impervious surfaces	10	400
Buildings: maximum height <2 m	10	400
Forest	20	200
Buildings: 2 m⩽maximum height < 5 m	20	200
Buildings: 5 m⩽maximum height < 10 m	50	80
Buildings: maximum height ⩾10 m	450	0

**Table 2:** For each zone, the table presents the mean percentage decline of observed and expected heterozygosities and the mean number of individuals lost at generation 10 and 100, computed on the basis of 5 simulation runs

*Zone*			*Observed heterozygosity*				*Expected heterozygosity*				*Number of individuals*			
			*Generation 10*		*Generation 100*		*Generation 10*		*Generation 100*		*Generation 10*		*Generation 100*	
			*Normal*	*Reduced*	*Normal*	*Reduced*	*Normal*	*Reduced*	*Normal*	*Reduced*	*Normal*	*Reduced*	*Normal*	*Reduced*
1	A	L	−7.3	−18.7	−23.7	−58.3	−4.6	−5.0	−19.5	−31.7	0.0	0.0	0.0	0.0
2	A	M	−15.1	−32.6	−28.6	−64.7	−3.6	−6.6	−14.0	−28.8	−0.8	−29.4	−0.8	−30.2
3	A	H	−35.9	−45.6	−65.6	−93.6	−7.3	−14.8	−30.6	−57.2	−29.3	−61.2	−40.2	−71.2
4	B	L	−7.2	−17.4	−18.5	−41.4	−3.0	−4.2	−12.1	−17.5	0.0	−1.2	0.0	−3.0
5	B	M	−8.7	−25.2	−19.5	−56.2	−3.5	−5.8	−11.9	−21.9	0.0	−3.0	0.0	−2.8
6	B	H	−36.2	−42.9	−66.1	−82.3	−6.0	−15.4	−21.1	−62.6	−18.2	−70.8	−25.8	−81.4
7	C	L	−6.0	−15.3	−16.9	−39.6	−3.4	−4.7	−13.6	−22.9	0.0	−0.8	0.0	−1.0
8	C	M	−14.6	−36.2	−32.2	−75.8	−3.9	−7.5	−14.6	−32.9	−5.0	−32.8	−3.7	−29.0
9	C	H	−30.1	−51.6	−60.9	−94.9	−6.6	−16.6	−28.1	−73.8	−17.2	−60.2	−25.5	−79.2
10	D	L	−5.9	−17.0	−19.2	−40.8	−3.6	−5.0	−17.2	−24.7	0.0	0.0	0.0	0.0
11	D	M	−12.8	−27.1	−34.3	−58.3	−4.4	−5.8	−18.1	−26.7	−3.0	−10.0	−3.0	−18.0
12	D	H	−38.0	−54.7	−82.1	−96.2	−6.4	−16.1	−26.8	−76.7	−17.8	−71.2	−28.7	−91.0

The names of the areas in the first column are indicated as follows: area number, transect and urbanisation level (L=low, M=medium and H=high). The indication ‘normal’ and ‘reduced’ are referring to the dispersal ability.
