# Supplementary material for: Persistence of butterfly populations in fragmented habitats along urban density gradients: motility helps
Source: Heredity (Edinb). 2017 Aug 9;119(5):328–38. doi: 10.1038/hdy.2017.40 (PMC5637364; doi:10.1038/hdy.2017.40)
Supplement: Supplementary Information [file hdy201740x1.doc]

**Supplementary information**

**Supp. Mat. 1:** List of parameters used with CDPOP.

We used the individual-based population genetics model software CDPOP 1.2.21 (Landguth and Cushman, 2010) to simulate the evolution of genotypes (500 SNPs) over 100 generations. The parameters used for these simulations are presented in the table below.

| agefilename | N |  | lmbda | 300 |
| --- | --- | --- | --- | --- |
| mcruns | 5 |  | Femalepercent | 50 |
| looptime | 100 |  | EqualsexratioBirth | N |
| nthfile_choice | sequence |  | birthmortperc | 99 |
| nthfile_seq | 1 |  | agemortperc | 95 |
| matemoveno | 1 |  | muterate | 0.0005 |
| matemovethresh | 4000 |  | mutationtype | random |
| sexans | Y |  | loci | 500 |
| Freplace | Y |  | intgenesans | random |
| Mreplace | Y |  | allefreqfilename | random |
| philopatry | N |  | alleles | 2 |
| multiple_paternity | Y |  | mtdna | N |
| selfans | N |  | cdevolveans | N |
| reproage | 0 |  | cdinfect | N |
| Fdispmoveno | 1 |  | cdclimate | N |
| Fdispmovethresh | 4000 |  | Edmatans | N |
| Mdispmoveno | 1 |  | gendmatans | N |
| Mdispmovethresh | 1350 |  | gridformat | cdpop |
| offno | 2 |  |  |  |

**Supp. Mat. 2**: ANOVA tables

In order to compare the genetic diversity and number of individuals remaining in each zone at the end of the simulations, we used the 5 simulation runs to compute a one-way ANOVA between the last values (generation 100) of H_obs_ (resp. H_exp_ and N) in each of the twelve zones (i.e. 12 groups, 5 measures per groups). The ANOVA tables obtained with the function *anova1* in Matlab R2014b are presented below.

**Supp. Mat. 3**: Scheffé tests

Post-hoc testing was performed using Scheffé test in order to highlight the significant differences between the twelve areas when considering the values reached at generation 100. The computations were performed using the function *multcompare* in Matlab R2014b software. The p-values obtained for each pairwise comparison are presented below. The significant ones (< 0.05) are highlighted in grey. The names of the areas in the first column are indicated as follows: area number, transect, urbanisation level (L=low, M=medium, H=high).
